# Is sitting invisible? Exploring how people mentally represent sitting

**DOI:** 10.1186/s12966-019-0851-0

**Published:** 2019-10-12

**Authors:** Benjamin Gardner, Stuart Flint, Amanda L. Rebar, Stephen Dewitt, Sahana K. Quail, Helen Whall, Lee Smith

**Affiliations:** 1 Department of Psychology, Institute of Psychiatry, Psychology and Neuroscience, King’s College London, Guy’s Campus, London, SE1 1UL UK; 2 Carnegie School of Sport, Leeds Beckett University, Leeds, UK; 3 Physical Activity Research Group, Appleton Institute, School of Health, Medical, and Applied Sciences, Central Queensland University, Rockhampton, Australia; 4 Department of Experimental Psychology, Division of Psychology of Language Sciences, University College London, London, UK; 5 The Cambridge Centre for Sport and Exercise Sciences, Department of Life Sciences, Anglia Ruskin University, Cambridge, UK

**Keywords:** Sedentary behaviour, Sitting, Standing, Cognition, Action identification, Psychology, Office workers, Experimental

## Abstract

**Background:**

Growing evidence suggests that prolonged uninterrupted sitting can be detrimental to health. Much sedentary behaviour research is reliant on self-reports of sitting time, and sitting-reduction interventions often focus on reducing motivation to sit. These approaches assume that people are consciously aware of their sitting time. Drawing on Action Identification Theory, this paper argues that people rarely identify the act of sitting as ‘sitting’ per se, and instead view it as an incidental component of more meaningful and purposeful typically-seated activities.

**Methods:**

Studies 1 and 2 explored whether people mentioned sitting in written descriptions of actions. Studies 3–5 compared preferences for labelling a typically desk-based activity as ‘sitting’ versus alternative action identities. Studies 6 and 7 used card-sort tasks to indirectly assess the prioritisation of ‘sitting’ relative to other action descriptions when identifying similar actions.

**Results:**

Participants rarely spontaneously mentioned sitting when describing actions (Studies 1–2), and when assigning action labels to a seated activity, tended to offer descriptions based on higher-order goals and consequences of action, rather than sitting or other procedural elements (Studies 3–5). Participants primarily identified similarities in actions based not on sitting, but on activities performed while seated (e.g. reading; Studies 6–7).

**Conclusion:**

‘Sitting’ is a less accessible cognitive representation of seated activities than are representations based on the purpose and implications of seated action. Findings suggest that self-report measures should focus on time spent in seated activities, rather than attempting to measure sitting time via direct recall. From an intervention perspective, findings speak to the importance of targeting behaviours that entail sitting, and of raising awareness of sitting as a potential precursor to attempting to reduce sitting time.

## Background

Prolonged sitting has been linked to adverse mental and physical health, and premature death [1–5]. This has spurred policy and research interest. National guidelines assert the importance of limiting sitting time [6–8]. Researchers have sought to describe and identify determinants of sitting patterns, often based on self-reported sitting [9, 10]. Various interventions have been trialled, many focusing on challenging motivation to sit [11]. Such research assumes that people are aware of their sitting, can reliably reflect on it and wish to reduce it. This paper questions such assumptions. We argue that people mentally represent sitting not as a purposeful act, but rather an incidental by-product of pursuing more meaningful actions.

Action Identification Theory [12] describes how people assign identities to behaviours: reading this paper, for example, could be identified as ‘reading a research report’ or ‘moving my eyes’. Action identities are hierarchically structured: higher levels capture general understandings of *why* an action is done, and lower levels represent more concrete details of *how* action is done. Levels of representation are relative; ‘reading a research report’ is a higher-level identity than ‘moving my eyes’, but lower-level than ‘learning about new research’. Action identities generate and sustain action, and higher-level identities, which reveal the purpose and likely consequences of action, tend to dominate because they offer optimal guides for action [12]. Assuming the present paper is being read to attain a desired goal (e.g. to learn about new research), for example, ‘reading a research report’ allows more effective implementation and monitoring of progress towards the goal than does ‘moving my eyes’. People thus mentally ‘chunk’ instrumental actions into higher-order action units. Action identification is a dynamic process, and people adopt lower-level identities where pursuit of the higher-level action is disrupted. For example, if the reader drops her glasses, a more procedurally-focused identity (‘picking up my glasses’) will temporarily dominate until recovery of and reversion to the higher-level identity (‘reading a research report’) is achieved.

Our thesis is that people rarely conceive of sitting as ‘sitting’, and instead assign higher-order action labels that convey the meaning of activities performed while sitting. That is, sitting is ‘invisible’; people seldom view ‘sitting’ as the purpose, nor do they value it as an outcome, of seated activity. If asked what they were doing, an office worker sitting at their desk would likely offer a description oriented in work-related goal pursuit (e.g. ‘working’), to which sitting is usually subservient [13]. People adopt lower-level identities for difficult or novel actions [12], but sitting is a simple and familiar act [9]. Documenting how people think about sitting could offer new avenues for understanding and reducing sedentary behaviour.

Action representations can be elicited in various ways, such as eliciting descriptions of actions, directly assessing preferences for one action identity over others, or indirectly assessing the prioritisation of identities in categorisation tasks [12, 14, 15]. We used various methods to assess how people mentally represent sitting. Studies 1 and 2 explored whether people mentioned sitting when freely describing their own and others’ actions. Studies 3–5 descriptively analysed preferences for labelling sitting as ‘sitting’ versus other action identities. Studies 6 and 7 used card-sort tasks to document the accessibility of ‘sitting’, relative to alternative action labels, when categorising similar actions.

## Study 1

This study investigated the accessibility of ‘sitting’ as an action representation by documenting the frequency with which people mentioned sitting when recalling autobiographical events. To identify whether people were inattentive to sitting per se, or to postural information more broadly, we also recorded mentions of standing. We predicted that:

Hypothesis 1: *When recalling previous events, people will not mention sitting or standing*.

We assumed a failure to mention sitting or standing would reflect that ‘sitting’ and ‘standing’ are not dominant representations, so are less cognitively accessible and unlikely to be elicited [16]. Support for our hypothesis could alternatively reflect a failure to encode postural information into memory. To explore this, we assessed the clarity of each recollection, and of specific aspects, including whether the participant was sitting or standing and location, others present, time, and clothes worn. The latter was included because we expected that, like posture, clothes worn would not be central to the meaning of the event.

### Method

#### Participants and procedure

Adults recruited from a UK-based online recruitment platform [17] were paid £1 (~US$1.30) to complete a task involving describing autobiographical events. Eligibility criteria were age (≥18 years), and English as first language. Of 178 adults that began the task, 28 did not complete it, and four were ineligible. The final sample comprised 146 participants (117 [80%] female; age 18-70y, mean = 34).

#### Data collection and analysis

Participants were asked to describe in as much detail as possible “*three recent experiences … things that you have done, or have happened to you, within the last three months”*. For each event, they also reported recency (today, yesterday, last week, a few weeks ago, last month, a few months ago), and clarity of the overall memory (‘my memory of this event involves [1 = little or no, 7 = a lot of] visual detail’) and of discrete aspects (‘my memory for [the time of day when / how many people were present when / the location in which / the clothes I was wearing when / whether I was standing or sitting down when] this event took place’ [1 = is vague, 7 = is clear/distinct]).

Verbs within each description were categorised into sitting (e.g., ‘sit’, ‘seated’, ‘sat’), standing (e.g., ‘stand’, ‘standing’, ‘stood’) or discrete other actions (e.g. ‘shopping’). We descriptively analysed the frequency of verbs and each action. Clarity ratings were nested within person and within events, so intraclass correlations were calculated for descriptive purposes, and comparisons tested using planned comparisons in multilevel models [18].

### Results and discussion

In total, 2445 verbs were coded within the 438 descriptions (mean 5.58 verbs per description, SD = 4.19), which were of events that typically occurred ‘a few weeks ago’ or more recently (*n* = 186). The verb frequency intraclass correlation (.64) indicated within-participant consistency in the number of actions recounted per event.

Contrary to Hypothesis 1, sitting was mentioned in 23 (5%) and standing in 5 descriptions (1%). The most common ‘other’ activity (‘going to [a destination]’) featured in 178 descriptions (41%).

Recollected events were rated as visually clear (minimum per-event mean = 4.36, SD = 1.38; see Additional file 1). Intraclass correlations revealed that 33% of variability in overall visual clarity was explained at the event level and 25% at the person level, suggesting consistency in clarity within participants and across events. Across events, participants reported clear memory of the time, location and others present (minimum per-event mean = 6.07, SD = 1.40), and clearer recollection of whether sitting or standing than of the overall event (z = 14.12, *p* < .01), or the clothes worn (z = 9.64, *p* < .01). There were no differences between perceived clarity of sitting or standing and event location (z = − 1.73, *p* = .48), presence of others (z = 1.24, *p* = .80), or time (z = − 2.55, *p* = .11).

Participants rarely mentioned sitting or standing when describing past events, though when prompted, remembered whether they were sitting or standing. This suggests that, while people committed postural information to memory, descriptions focused on the purpose or consequences of actions – for example, to arrive at a destination – rather than subservient elements such as posture.

Although participants expressed confidence in recalling posture, other elements of the event may have provided cues to posture; visiting the cinema, for example, entails sitting. Autobiographical events are inherently idiosyncratic, and variation in duration and number of discrete actions within such events may have influenced the likelihood of posture being recounted. We were unable to verify the accuracy of recollections; autobiographical memories are often inaccurate or incomplete [19]. Study 2 explored mentions of sitting in descriptions of a standardised set of stimuli.

## Study 2

Action identification principles apply equally to one’s own and others’ behaviours; people tend to identify others’ actions by inferring the actors’ thoughts, emotions and intentions [20]. This study assessed the salience of posture in participants’ descriptions of photos of others performing activities while sitting or standing. Following Study 1, we predicted:

Hypothesis 2: *When describing others’ actions, people will be less likely to mention sitting or standing than other action identities*.

### Method

#### Participants and procedure

Participants were directed to an online task via an email circular to staff and students in a UK university, an advertisement on an undergraduate research participation pool system, and a social media post. Undergraduates received course credits, but no other incentives were provided. Eligibility criteria were as in Study 1.

The task featured sixteen photos of individuals sitting or standing, for each of which participants had to write a description. Three illustrative examples, spanning different identification levels, were provided (e.g. ‘people watching a live band’, ‘people putting their hands in the air’). Of 122 people who began the task, 19 were ineligible, and 33 did not complete, leaving a final sample of 70 (58 [83%] female; aged 18–57 years, mean = 27).

#### Materials

Photos were selected from a public photo-sharing website [21] where they met the following criteria: colour, no obvious editing, depicting at least one person with open eyes and unambiguously standing or sitting while performing another activity. Photos depicting famous people, babies, more than 10 people, or nudity were excluded. Eight photos depicted sitting and eight standing, of which three showed ‘active standing’ (i.e., walking, running).

#### Data collection and analysis

Participants were instructed to *“describe the action or actions that you see in [each] image, in no more than one sentence”*. Verbs were coded and categorised as in Study 1. To ensure the most salient action identity was elicited, where descriptions exceeded one sentence, only the first sentence was coded. Factually inaccurate or verb-free descriptions (e.g. ‘man in a park’) were excluded. Paired-samples t-tests compared, for each photo, the proportion of responses citing sitting or standing versus the most commonly-elicited ‘other’ activity.

### Results and discussion

Both sitting (t = 4.65, *p* = .002) and standing (t = 5.03, p = .002) were less commonly elicited than other action identities, supporting Hypothesis 2. For seven of eight sitting photos, other identities dominated: the proportion of citations of sitting across photos ranged from 3 to 37%, whereas alternative action citations ranged from 26 to 100%. Where sitting was mentioned, it was commonly alongside other action identities (e.g. ‘sitting and drawing’). Similarly, standing was less frequently cited for seven of eight photos, and was typically described with other actions (e.g. ‘standing watching football’). These findings support our assumption that ‘sitting’ and ‘standing’ are not salient representations.

Studies 1 and 2 assessed mental representations within freely-generated descriptions. Participants may have excluded postural information because they perceived it to have insufficient communicative value; nobody mentioned ‘living’ or ‘breathing’ in either study, for example. Studies 3–5 circumvented this problem by examining explicit preferences for posture-based action representations.

## Study 3

This study directly assessed preferences for labelling activities as ‘sitting’ (or ‘standing’) versus an alternative action identity. We predicted that:

Hypothesis 3: *When describing seated or standing activities, people will be unlikely to assign an action identity based on sitting or standing.*

### Method

#### Participants, procedure and materials

225 adults recruited via a US-based online research recruitment platform [22] completed an online task. Age (≥18 years) was the only eligibility criterion. Due to researcher error, no demographic information was collected. The survey comprised five items, based on the Behavioural Identification Form (BIF), a validated action identification tool [23]. Each item presented an action (e.g. ‘locking the front door’), and two valid alternative action descriptions, one based on more concrete elements (i.e. lower-level identity; e.g. ‘putting the key in the lock’), and one addressing the presumed purpose of action (higher-level; ‘securing the house’ [23]). Participants were required to ‘*choose the identification that best describes the behaviour for you*’. Three filler items were randomly selected from the BIF. The two focal items related to sitting (‘using the office computer’; response options: ‘sitting down’ vs ‘getting work done’) and standing (‘getting out of bed’; ‘standing up’ vs ‘starting the day’).

#### Analysis

Chi-squared goodness-of-fit tests assessed preferred descriptions for each action.

### Results and discussion

Most participants (168; 75%) preferred ‘getting work done’ over ‘sitting down’ as a label for ‘using the office computer’ (χ^2^ = 54.76, *p* < .001), and 177 (79%) preferred ‘starting the day’ over ‘standing up’ as a label for ‘getting out of bed’ (χ^2^ = 73.96, p < .001). Hypothesis 3 was supported. This cannot be attributed to postural information lacking salience, because sitting and standing were explicitly offered as possible action labels.

Higher-level action identities were preferred for the three BIF items (maximum *p* = .002). Our findings imply that, compared to alternative action identities, ‘sitting’ was deemed less applicable because it does not convey the purpose or implications of seated activities. We did not however directly assess the meaningfulness of sitting. Study 4 tested whether sitting and standing are indeed perceived as mechanistic rather than purposeful action identities.

## Study 4

This study investigated the level of abstraction at which people portray ‘sitting’ relative to alternative action labels for an archetypal sedentary behaviour (i.e. desk-based activity [24]). Following Wegner and colleagues [15], participants rated the extent to which action identities described desk-based activity, and factor analysis identified response clusters corresponding to identification levels. We argue that sitting is primarily seen as instrumental in pursuing more meaningful actions, so predicted that:

Hypothesis 4: *People will portray the act of ‘sitting’ at the same level of abstraction as other procedural action identities.*

### Method

#### Participants, procedure and materials

Office workers were recruited via a UK-based online recruitment platform [17]. Eligibility criteria were age (≥18 years) and, to ensure personal relevance of the focal action, working full-time in office-based employment. Participants completed an online task in which they were rated how well each of 20 activities (including ‘sitting’) accurately described *‘what you personally do at a desk’* (1 = not well at all, 7 = extremely well [15]). The 20 activities were independently generated by a separate panel of eight university-based office workers.

Of 150 people who completed the survey, 11 did not meet eligibility criteria. Our final sample comprised 139 participants (81 [58%] female; age range 22–71 years, mean = 39).

#### Analysis

Direct oblimin principal component analysis identified discrete factors underlying responses, with observed eigenvalues compared to randomly-generated thresholds [25]. Only items that loaded at ≥.40 were deemed indicative of factors. Bartlett’s test of sphericity was significant (χ^2^ (190) = 1165.83, *p* < .001), and sampling adequacy was high (KMO = .82), indicating acceptability of analysis.

### Results and discussion

All 20 action were typically viewed as descriptive (range of means: 4.12–6.24), and ‘sitting’ was particularly descriptive (mean = 5.88, SD = 1.40). Four factors were extracted (see Table 1). The first, which explained most variance in responses, appeared to capture procedural actions (e.g. ‘typing’, ‘looking at the monitor’, ‘pressing buttons’), and included ‘sitting’. The second factor related to meeting work responsibilities (e.g. ‘working’, ‘doing my job’). The third factor, except one item (‘moving my hands’), related to economic implications of work (e.g., ‘contributing to the economy’, ‘earning money’), and the fourth to information processing (e.g., ‘organising information’). These data support Hypothesis 4 by suggesting that sitting was viewed as one of several procedural (i.e. relatively low-level) desk-based activities, distinct from higher-level identities that convey the broader social or organisational functions of such activities.

**Table 1:** Study 4: Principal component analysis of possible descriptors of ‘what I do at a desk’

	*Mean applicability rating (SD)*	*Factor loadings (≥.40)*			
*Action identity*		*Factor 1*	*Factor 2*	*Factor 3*	*Factor 4*
Looking at the monitor	5.76 (1.36)	.78			
Sitting	5.88 (1.40)	.68			
Pressing buttons	4.97 (1.81)	.64			
Moving the cursor	5.35 (1.69)	.59			
Using the internet	5.29 (1.52)	.54			
Using my computer	6.24 (1.17)	.50			
Typing	5.48 (1.35)	.47			
Reading information	5.53 (1.27)	.40			
Working	6.06 (1.14)		.83		
Doing my job	5.98 (1.07)		.83		
Getting my work done	5.83 (1.04)		.72		
Contributing to the economy	4.12 (1.73)			−.59	
Moving my hands	4.91 (1.85)			−.55	
Honouring my employment contract	5.38 (1.62)			−.48	
Earning money	5.84 (1.21)			−.45	
Furthering my career	4.30 (1.61)			−.43	
Organising information	5.42 (1.45)				−.74
Processing information	5.78 (1.19)				−.62
Making progress	5.16 (1.34)				−.42
Earning a living	5.96 (1.15)				
Eigenvalue		6.13	2.60	1.58	1.38
% variance explained		30.64	13.01	7.91	6.88
Inter-factor correlations:					
Factor 1			.09	−.30	−.33
Factor 2				−.26	−.23
Factor 3					.28

Although mean scores indicated that ‘sitting’ was viewed as descriptive of desk-based activity, the task did not explicitly assess the priority of ‘sitting’ as an action representation relative to the nineteen alternative action identities. Study 5 explored preferences for identifying desk-based action as ‘sitting’ compared to alternative action identities.

## Study 5

Study 3 documented, in a binary choice task, preferences for describing desk-based activity using labels reflecting work-related goals (i.e. ‘getting work done’), rather than as ‘sitting’, which was represented in Study 4 as a mechanistic action. Study 5 extended these findings by examining the prioritisation of ‘sitting’ versus multiple alternative actions, drawn from the four factors extracted in Study 4. Office workers ranked action identities according to perceived descriptiveness of desk-based activity. We predicted that:

Hypothesis 5: *People will rate ‘sitting’ as less descriptive than identities that focus on the purpose and consequences of desk-based activity.*

### Method

#### Participants, procedure and materials

One-hundred and forty-nine office workers (77 [52%] female, aged 18–68 years, mean = 38) were recruited via a UK-based online recruitment site [17]. Eligibility criteria were age (≥18 years), and employed full- or part-time in professional, managerial or administrative roles. Participants were paid £0.85 (~US$1.10) on completing an online task in which they ranked 10 randomly-ordered action identities according to *“how well they describe what you personally do at your desk”* (1 = most, 10 = least descriptive). Action identities were a subset of the 20 used in Study 4, capturing each of the factors extracted in Study 4 (see Table 2).

**Table 2:** Study 5: Mean and median descriptiveness rankings of desk-based action identities

*Action identity*	*Factor label (from Study 4)*	*Mean rank (SD)*	*Median rank*
Working	Work responsibilities	3.78 (2.71)	3
Doing my job	Work responsibilities	4.27 (2.77)	3
Getting my work done	Work responsibilities	4.38 (2.51)	4
Using my computer	Procedural activities	4.67 (2.40)	5
Processing information	Information processing	5.24 (2.39)	5
Organising information	Information processing	5.91 (2.47)	6
Typing	Procedural activities	6.28 (2.35)	7
Looking at my monitor	Procedural activities	6.50 (2.65)	7
Sitting	Procedural activities	6.72 (3.63)	8
Honouring my employment contract	Economic impact	7.25 (2.84)	8

#### Analysis

Friedman’s ANOVA with follow-up Wilcoxon tests compared mean rankings for ‘sitting’ versus identities deemed most representative of the three non-procedural factors from Study 4 (‘working’, ‘honouring my employment contract’, ‘organising information’). Representativeness of the latter three items was based on higher loadings and descriptiveness ratings in Study 4.

### Results and discussion

The activity typically ranked as most descriptive was ‘working’ (mean 3.78, median 3; Table 2), and rankings differed across items (*p* < .001). ‘Sitting’ was assigned a low rank (mean 6.72, median 8), and was viewed as less descriptive than ‘working’ (mean 3.78, median 3; T = 1.01, p < .001), but not ‘organising information’ (mean 5.91, median 6; T = 0.34, *p* = .13) nor ‘honouring my employment contract’ (mean 7.25, median 8; T = − 0.24, *p* = .70). Hypothesis 5 was partially supported.

Taken together with Studies 3 and 4, findings indicate that ‘sitting’, while seen as highly applicable to desk-based activity, lacks priority as a representation of such activity. Preferred action identities related to work responsibilities (e.g. ‘working’, ‘doing my job’).

Studies 3–5 directly assessed action representations and may have primed responses; participants may not have identified desk-based activity as ‘sitting’, or indeed assigned any other lower-level identity, had these identities not been made salient by data collection materials. Studies 6 and 7 adopted indirect methods to elicit action representations.

## Study 6

This study conceptually replicated and extended Studies 3 and 5 by assessing indirectly the accessibility of ‘sitting’ and ‘standing’ compared to alternative identity labels. Participants viewed three photos, two of which depicted a person sitting (or standing), and two depicted a person engaging in a presumed higher-level action (e.g. reading) and were asked to select two photos depicting the same action. We predicted that:

Hypothesis 6: *People will identify similarities between actions based on common higher-level action identities rather than ‘sitting’ or ‘standing’.*

### Method

#### Participants, procedure and materials

Adults recruited via a US-based crowdsourcing website [22] were paid ~£0.30 (US$0.40) on completing a brief online task involving selecting two of three photos depicting people engaging in the same activity. Age (≥18 years) was the only eligibility criterion. Colour photographs were selected from a photo-sharing website [21] on the basis that they depicted one adult, unambiguously standing or sitting and performing another activity. Participants were randomly assigned to view one of four sets of three photos (see Table 3). Each set featured two photos depicting a person performing different higher-level actions (e.g. eating, smoking) in the same posture (sitting or standing), and two showing people doing the same higher-level action in different postures.

**Table 3:** Study 6: Frequency with which action similarities identified

*Set*	*N*	*Photo*	*Posture*	*Higher-level action*	*Higher-level pairings (photos A-B), N (%)*	*Posture-based pairings (photos A-C), N (%)*	*χ* ^*2*^
1	67				65 (97.0%)	2 (3.0%)	59.24***
		A	Standing	Talking on phone			
		B	Sitting	Talking on phone			
		C	Standing	Painting			
2	62				61 (98.4%)	1 (1.6%)	58.07***
		A	Sitting	Playing guitar			
		B	Standing	Playing guitar			
		C	Sitting	Looking at laptop			
3	56				52 (92.9%)	4 (7.1%)	41.14***
		A	Sitting	Reading			
		B	Standing	Reading			
		C	Sitting	Taking a ‘selfie’			
4	73				60 (82.2%)	13 (17.8%)	30.26***
		A	Sitting	Eating			
		B	Standing	Eating			
		C	Sitting	Smoking			
*Total*	258				238 (92.2%)	20 (7.8%)	184.20***

***p < .001

Of 268 participants, one completed the task incorrectly, and nine chose ineligible pairings (e.g. ‘sitting and eating’, ‘standing and reading’), leaving a final sample of 258 (112 [43%] female; age range 18–71 years, mean = 36).

#### Analysis

Chi-squared tests compared the frequency with which photos were paired according to posture versus higher-level actions.

### Results and discussion

Across all photo sets, participants were more likely to perceive similarities based on higher-level actions (*N* = 238; 92.2%) than on sitting or standing (*N* = 20; 7.8%; χ^2^ = 184.20, *p* < .001).^1^ The same pattern was observed within each set of photos (all p’s < .001). Supporting Hypothesis 6, participants were consistently more attentive to higher-level identities than to sitting or standing when identifying similarities between actions. The binary nature of the task, however, precludes assessment of the priority assigned to ‘sitting’ or ‘standing’ relative to multiple alternative identities.

## Study 7

This study extended Study 6, via a card-sort task involving identifying multiple action similarities within photos of others, to assess the priority of ‘sitting’ and ‘standing’ among other action identities. Participants were incentivised to identify as many pairings of people ‘doing the same thing’ as possible within a set of 12 photos with multiple similarities. We assumed that the order in which similar actions were identified reflected cognitive accessibility (16), such that people would first attend to similarities corresponding to dominant action identities, and any similarities in posture would only be identified in later pairings. Thus, we predicted that:

Hypothesis 7: *People will (a) be unlikely to identify similarities between actions based on ‘sitting’ or ‘standing’, and (b) the first similarity identified will not be based on ‘sitting’ or ‘standing’.*

### Method

#### Participants, procedure and materials

Adults were recruited via a UK online platform [17] to complete an online task that involved selecting, from a set of 12 photos, pairs depicting people doing the same action. Age (≥18 years) was the only eligibility criterion. Photos were created especially for the study, using four models (two female), and were verified in a pilot study of 40 participants to be affectively neutral on both valence and activation [26, 27]. Six photos depicted sitting, and six standing. Five other activities were depicted across the photos: painting, reading, talking on the phone, taking a ‘selfie’, and using a tablet computer (Additional file 1). To mask the study purpose, the frequency with which actions were depicted varied, with one action (talking on phone) featuring in only one photo, and one (painting) in four photos.

Participants were asked to select a pair of photos and describe in free-text ‘*the thing that both of the people in these two photos are doing’*. After identifying five pairings, participants could exit the task at any point. Participants were paid £1 (~US$1.30) on completion, and to incentivise continuation, an additional £25 (~US$33) cash was offered to the person who identified most valid pairings.

Of 165 participants that began the survey, 14 did not complete it, and three were excluded because their free-text responses were not written in English. The final sample comprised 148 participants (82 [55%] female; aged 18–77 years, mean = 32).

#### Analysis

Written descriptions were coded to identify verbs, which were coded and categorised as in Study 1. Inaccurate descriptions, or descriptions lacking verbs and not identifiable as relating to action (e.g. ‘same brushes in background’), were treated as invalid. Where multiple verbs were cited, only the first was coded. Data were descriptively analysed.

### Results and discussion

Participants selected an average of 6.5 photo pairings (SD = 1.47, range = 5–9, median = 6). Of 951 written descriptions, 4 were invalid. Contrary to Hypothesis 7a, 81 participants (55%) identified similarities based on sitting or standing at least once. Posture was mentioned in 255 (27%) descriptions (137 sitting, 118 standing).

Descriptions of the first identified pairing showed that only 22 (15%) referred to posture (13 sitting, 9 standing), offering some support for Hypothesis 7b. Among the 81 participants who paired photos according to sitting or standing, posture was typically identified within the third pairing (mean rank = 3.19; range 1–7, median = 3).

While most people accounted for sitting or standing when identifying similarities between actions, ‘sitting’ and ‘standing’ were of lesser priority than alternative action labels.

## General discussion

Any behaviour can be labelled in multiple ways; a person on a bus, for example, may view her action as ‘commuting’ or ‘sitting’ [12]. Our participants typically represented seated episodes according to the activities undertaken while sitting and viewed ‘sitting’ mostly as a mechanistic description of how such activities are enacted [12]. Participants showed awareness of sitting, suggesting that sitting is not wholly ‘invisible’, but rather a deprioritized, less accessible representation of seated activity. Similar results were observed for representations of standing, indicating a broader deprioritization of postural information in cognitive representations of everyday activities. Vallacher and Wegner [28] distinguish between ‘behaviour’, which describes movement, and ‘action’, which describes *purposeful* movement. From this perspective, our results suggest that while sitting is a behaviour of interest to researchers, it is rarely an *action* from the actor’s perspective, instead being represented as a by-product of engaging in more meaningful seated actions. These findings may have important measurement and intervention implications.

Although reliable objective measures of sitting time are available [29], much empirical research into sitting time relies on self-report [30], which assumes that people can accurately reflect on sitting. Yet, in Study 1, sitting was rarely cited in verbal descriptions of autobiographical events, suggesting that ‘sitting’ may not be reliably encoded into or retrieved from memory. Although participants were confident in recollecting their posture during these events, we were unable to assess the accuracy of their recollections. It is well-documented that people misreport sitting time. One study, for example, found that a direct self-report item (‘how long per working day did you spend sitting?’) underestimated mean monitor-assessed daily sitting time by 204mins [31] (see too [32]). While this may be partly attributable to self-presentation biases [33], self-report accuracy may also be limited because people do not view episodes of seated activity as ‘sitting’. Directly reporting sitting time thus requires mental calculations to translate time spent in meaningful seated activities (e.g. ‘watching TV’) into sitting time [34], a process susceptible to error and bias [35]. This would explain why indirect measures, which infer sitting time from time spent in typically-seated activities, typically yield more accurate responses than direct measures [36]. Further work is needed to test whether viewing seated action as ‘sitting’ improves self-report accuracy. Nonetheless, where objective sedentary behaviour assessment methods are unavailable, we recommend that researchers adopt indirect self-report measures, which place the onus for sitting time estimation on the researcher rather than the participant.

For desk-based activity at least, participants viewed ‘sitting’, alongside ‘typing’ and ‘looking at the monitor’, as a finer-grained procedural component incurred by completing work tasks. Action Identification Theory proposes that people mentally represent actions according to why and with what effect they are done, because such representations (e.g. ‘working’) convey information to guide goal-directed activity in a way that representations detailing how action is done do not [12]. By implication, our data suggest people sit not for the purpose of sitting, but because they are motivated to perform activities that entails sitting. This qualifies research efforts that seek to understand motivations to sit [10]. For example, in one study, children who expressed preferences for seated tasks (e.g. playing video games) lost less weight following a sedentary reduction intervention [37]. The authors concluded that ‘the *motivation to be sedentary* limits the effects of reducing sedentary behaviour on weight change’ (p1; emphasis added). Our results, however, suggest that the motivation *to engage in activities that involve sitting* accounted for such effects. Attempts to assess motivation to sit – rather than to pursue seated activities – via questionnaire methods risk capturing cognitions generated in response to questionnaire items, rather than pre-existing cognitions [38].

Intervention developers should acknowledge that sitting per se is rarely consciously motivated. Interventions might seek to increase awareness of sitting patterns as a precursor to reduction. People often express surprise upon realizing their sitting time [39], suggesting they do not consciously attend to it. Raising awareness, by for example objectively monitoring and providing retrospective feedback on time spent sitting, can motivate people to reduce their sitting time [40–42]. Interventions might also reduce sitting indirectly, by targeting actions that incur sitting. This will require acknowledging the meaning, purpose and function of seated actions, and promoting sitting reduction in a way that impacts minimally on pursuit of such actions or desired consequences [43, 44]. For example, for many older adults, seated activities serve important social functions (e.g. meeting friends for coffee), confer cognitive benefits (e.g. doing crosswords), or are instrumental to relaxation (e.g. watching TV [43]). Similarly, office workers typically sit to complete work tasks [13], and so software that deactivates computer workstations at regular intervals to compel breaks from sitting disrupted participants’ workflow can prompt frustration [45] (see too [46]). Environmental modification strategies, such as adjustable sit-stand workstations, which permit normally-seated tasks while standing or moving, show acceptability and can reduce sitting [11, 47, 48].

Limitations must be acknowledged. The demographic diversity and representativeness of users of the online platforms from which we recruited has been questioned [49]. However, we have no reason to expect that demographics contributed to observed effects. Moreover, action identification is a dynamic process, such that lower-level representations such as ‘sitting’ may be prioritised in response to contextual changes, such as when no seat is available. However, we would not expect ‘sitting’ to become a dominant action representation other than in response to momentary contextual disruptions. Additionally, some people typically identify actions at finer-grained levels of analysis rather than according to their broader meaning [23]. ‘Sitting’ may therefore be a more prominent representation for some people. However, the lack of prioritisation of ‘sitting’ as an action identity appeared to be a strong, robust effect: in Study 3, 75% of participants expressed a preference for labelling seated and standing activities according to higher-level meanings rather than posture, and in Study 6, 92% identified similarities between photos based on purposeful acts rather than posture. Any effects of individual differences on identity preferences are likely to have been small.

Our studies assumed that people assign single-action labels to activities (e.g. ‘drawing’ [12]). Yet, in free-text descriptions, participants used multi-action labels (‘sitting and drawing’), suggesting that they hold representations that combine multiple concurrent activities. More recent theorising proposes that people store comprehensive representations incorporating information on multiple actions, alongside sensory information, information about cognitions, affect and goals, and contexts [50, 51]. Nonetheless, our studies suggest that within such representations, ‘sitting’ may be less heavily weighted or meaningful.

## Conclusion

Much research tacitly assumes that sitting is a meaningful action. Our studies challenge this claim; people rarely represented seated activity as ‘sitting’, instead viewing it as an instrumental element of more meaningful activities performed while seated. Sedentary behaviour researchers should recognize that sitting is often not a motivated action, but rather is incurred by and subservient to other activities. Developing acceptable and effective sitting-reduction interventions may depend on reducing sitting in a way that respects the purpose and value that people assigned to seated actions.

## Supplementary information

**Additional file 1: Table S1.** Study 1: Perceived clarity of memories of autobiographical events.

**Additional file 2: Table S2.** Study 7: Description of stimuli.

## Data Availability

All datasets analysed within the current studies are available from the corresponding author on reasonable request.

## Abbreviations

BIF: Behavioural Identification Form
SD: Standard deviation
TV: Television

## Footnotes

1 We varied the task instructions, randomly assigning participants to be asked to identify people doing the same ‘behaviour’ (*n* = 70), ‘thing’ (*n* = 79), ‘activity’ (*n* = 57), or ‘action’ (*n* = 52). The same pattern of preferences for higher-order action similarities was found across all instruction variants (all p’s < .001). The same pattern of results also held regardless of task completion time, comparing those who paired photos below versus at or above median completion time (7.13 s; p’s < .001).
