# Supplementary material for: Is sitting invisible? Exploring how people mentally represent sitting
Source: Int J Behav Nutr Phys Act. 2019 Oct 12;16:85. doi: 10.1186/s12966-019-0851-0 (PMC6790031; doi:10.1186/s12966-019-0851-0)
Supplement: Supplementary file 1 — Additional file 1: Table S1. Study 1: Perceived clarity of memories of autobiographical events. [file 12966_2019_851_MOESM1_ESM.docx]

**Additional File 1. Supplementary Table 1, Study 1:** Perceived clarity of memories of autobiographical events

|  | *Event 1*  *Mean (SD)* | *Event 2*  *Mean (SD)* | *Event 3*  *Mean (SD)* |
| --- | --- | --- | --- |
| Overall visual clarity | 4.36 (1.38) | 4.51 (1.32) | 4.47 (1.38) |
| Time | 6.43 (1.05) | 6.23 (1.22) | 6.07 (1.40) |
| Presence of others | 6.52 (0.97) | 6.43 (0.99) | 6.27 (1.42) |
| Location | 6.85 (0.56) | 6.81 (0.54) | 6.61 (1.04) |
| Clothes worn | 5.31 (2.03) | 5.04 (2.10) | 4.71 (2.21) |
| Sitting/standing | 6.69 (0.82) | 6.50 (0.90) | 6.47 (1.16) |
