# Supplementary material for: Is sitting invisible? Exploring how people mentally represent sitting
Source: Int J Behav Nutr Phys Act. 2019 Oct 12;16:85. doi: 10.1186/s12966-019-0851-0 (PMC6790031; doi:10.1186/s12966-019-0851-0)
Supplement: Supplementary file 2 — Additional file 2: Table S2. Study 7: Description of stimuli. [file 12966_2019_851_MOESM2_ESM.docx]

**Additional File 2. Supplementary Table 2, Study 7**: Description of stimuli

| *Photo* | *Sitting or standing* | *Higher-level action* |
| --- | --- | --- |
| A | Standing | Taking ‘selfie’ |
| B | Sitting | Painting |
| C | Sitting | Reading |
| D | Sitting | Reading |
| E | Standing | Using a tablet computer |
| F | Sitting | Talking on phone |
| G | Standing | Painting |
| H | Standing | Using a tablet computer |
| I | Standing | Painting |
| J | Standing | Taking ‘selfie’ |
| K | Sitting | Using a tablet computer |
| L | Sitting | Painting |
